# No association between habitat, autogeny and genetics in Moroccan *Culex pipiens* populations

**DOI:** 10.1186/s13071-022-05469-3

**Published:** 2022-11-03

**Authors:** Soukaina Arich, Yuki Haba, Najlaa Assaid, Megan L. Fritz, Carolyn S. McBride, Mylène Weill, Hassan Taki, M’hammed Sarih, Pierrick Labbé

**Affiliations:** 1 Institut Des Sciences de L’Évolution de Montpellier, UMR 5554, CNRS-UM-IRD-EPHE, Université de Montpellier, Montpellier, Cedex 5 France; 2 Laboratory of Biology and Health, Faculty of Sciences Ben M’Sik, URAC34, Hassan II University of Casablanca, Casablanca, Morocco; 3 Laboratoire Des Maladies Vectorielles (LMV), Institut Pasteur du Maroc, Casablanca, Morocco; 4 Department of Ecology and Evolutionary Biology, Princeton University, Princeton, NJ 08544 USA; 5 Department of Entomology, University of Maryland, College Park, MD USA; 6 Institut Universitaire de France, 1 rue Descartes, 75231 Cedex 05 Paris, France; 7 Princeton Neuroscience Institute, Princeton University, Princeton, NJ 08544 USA

**Keywords:** Autogeny, *Culex*, Vector, Morocco

## Abstract

**Background:**

Mosquitoes of the *Culex pipiens* complex are found across the globe and are the focus of many research studies. Among the temperate species *C. pipiens* sensu stricto (s.s.), two forms are usually described: *molestus* and *pipiens*. These two forms are indistinguishable in terms of morphology but show behavioral and physiological differences that may have consequences for their associated epidemiology. The two forms are well defined in the northern part of the species distribution, where autogeny is strictly associated with the *molestus* form. However, whether the two remain distinct and show the characteristic differences in behavior is less clear in North Africa, at the southern edge of their range.

**Methods:**

The association between autogeny, as determined by ovarian dissection, and molecular forms, based on the CQ11 microsatellite marker, was studied in six Moroccan populations of *C. pipiens*.

**Results:**

An overall low prevalence of autogeny was found at three of the Moroccan regions studied, although it reached 17.5% in the Agadir population. The prevalence of form-specific CQ11 alleles was quite similar across all populations, with the *molestus* allele being rarer (approx. 15%), except in the Agadir population where it reached 43.3%. We found significant deficits in heterozygotes at the diagnostic CQ11 locus in three populations, but the three other populations showed no significant departure from panmixia, which is in line with the results of a retrospective analysis of the published data. More importantly, we found no association between the autogeny status and CQ11 genotypes, despite the many females analyzed.

**Conclusions:**

There was limited evidence for two discrete forms in Morocco, where individuals carrying *pipiens* and *molestus* alleles breed and mate in the same sites and are equally likely to be capable of autogeny. These observations are discussed in the epidemiological context of Morocco, where *C. pipiens* is the main vector of several arboviruses.

**Graphical Abstract:**

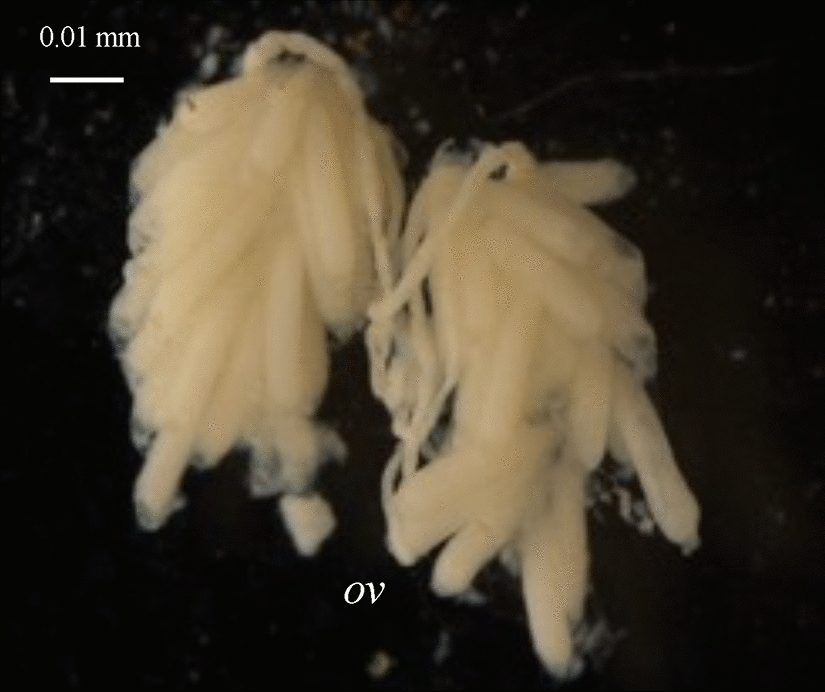

**Supplementary Information:**

The online version contains supplementary material available at 10.1186/s13071-022-05469-3.

## Background

*Culex (C.) pipiens* has been, and still is, the focus of attention of many biologists, vector and evolutionary biologists in particular, who study their vectorial capacity for various viruses and pathogens (e.g*. *[1–4]), their resistance to insecticides [5–10], their reproduction [11, 12] and/or their phylogenic origin [13–16]. *Culex pipiens* is one of the most geographically widespread species of mosquitoes. It is considered to be a species complex that includes *C. pipiens *sensu stricto (s.s.), *C. quinquefasciatus*, *C. australicus* and *C. globocoxitus* [2, 17]. While *C. australicus* and *C. globocoxitus* are restricted to Australia, *C. quinquefasciatus* and *C. pipiens *s.s. are spread across the globe, with the former in tropical/subtropical regions and the latter in temperate regions, although there are hybridization zones in the Americas and Asia [13].

The *C. pipiens *s.s. found in temperate regions is usually described as comprising two forms, *C. p. pipiens* (referred to as *pipiens* hereafter) and *C. p. molestus* (referred to as *molestus* hereafter), whose evolutionary relationships remain under debate (for a detailed review see [18]). They display indistinguishable morphologies, but show behavioral and physiological differences that greatly influence their vector competencies, including, for example, their intrinsic capacity to host and transmit viruses and pathogens [13].

The *molestus* form is usually described as subterranean, occupying environments with limited surface access, while the *pipiens* form lives above-ground. Both forms display adaptations to their typical environment, although it is probable that the *molestus* adaptations actually precede their colonization of the underground regions [18]: *molestus* mate in confined spaces (stenogamy), feed on mammals, including humans (mammophilia), and remain active during winter (homodynamic), while *pipiens* by contrast mate in open spaces (eurygamy), feed predominantly on birds (ornithophilic) and undergo winter diapause (heterodynamic) [19].

The most striking difference between these two forms lies in the capacity of *molestus* females to lay their first eggs without a blood meal, referred to as autogeny, while the *pipiens* females are usually described as being anautogenous. In anautogenous females, a blood meal is required to activate vitellogenesis, *i*.*e.* follicle development and deposition of yolk proteins, through signaling pathways that likely involve juvenile hormone, insulin-like peptide, ecdysone and target of rapamycin (TOR) [20–24]. In autogenous females, the same signaling pathways control egg maturation within the first few hours after emergence, without a blood meal [25]. Autogeny appears to be genetically encoded: while a nutrient-rich larval habitat allows autogenous females to lay a larger first clutch of eggs, they can produce eggs without a blood meal even in poor environments; conversely, anautogenous females cannot become autogenous even in a nutrient-rich site [22].

The situation is further complicated by the existence of a latitudinal gradient in hybridization between these two forms, from no hybridization at all in northern Europe, to limited gene flow in southern Europe [13, 18, 26]. This is particularly visible in the distribution of a marker flanking a microsatellite locus called CQ11 [27]. This marker (referred to as CQ11 hereafter) has two alleles, each specific to one of the forms found in northern Europe [14]. In southern Europe, while the *molestus* allele is largely dominant in belowground populations, above-ground populations harbor a mix of *molestus* and *pipiens* alleles, but with fewer heterozygotes than expected in a panmictic population [14, 28, 29].

Relatively less is known on the status of *C. pipiens* forms in North Africa. In the few studies available, two from Tunisia and one from Algeria, all populations appear to be well mixed, with both CQ11 alleles present, and heterozygote frequencies are similar to those expected for panmixia [30–32]. These observations support the presence of a latitudinal gradient [18] and suggest that North African *C. pipiens* populations are somewhat intermediate between the two northern extremes. Nevertheless, more data are needed to confirm the generality of this pattern.

More importantly, while the autogenous or anautogenous characters are strongly associated with the *molestus* and *pipiens* forms, respectively, in northern Europe, this association is less clear in the few studies available for the North African region of the species distribution. For example, in Egypt, most individuals are morphologically characterized as *molestus*, but only few females can lay eggs without a blood meal [33], and in Tunisia, autogeny is found in individuals carrying both *molestus* and *pipiens* CQ11 alleles [32]. Therefore, it remains unknown how, if at all, the CQ11 alleles are associated with autogeny in these mixed *C. pipiens* populations from North Africa.

Morocco presents a most suitable opportunity to address this question: similar to the situation in Tunisia and Algeria, a previous analysis showed that both alleles of the CQ11 marker segregate in natural populations, with many heterozygotes [34]. In the present study, we took advantage of this situation to assess the association between autogeny, revealed through ovarian dissection, and CQ11 genotypes.

### Methods

## Mosquito collections and identification

**Fig. 1:**
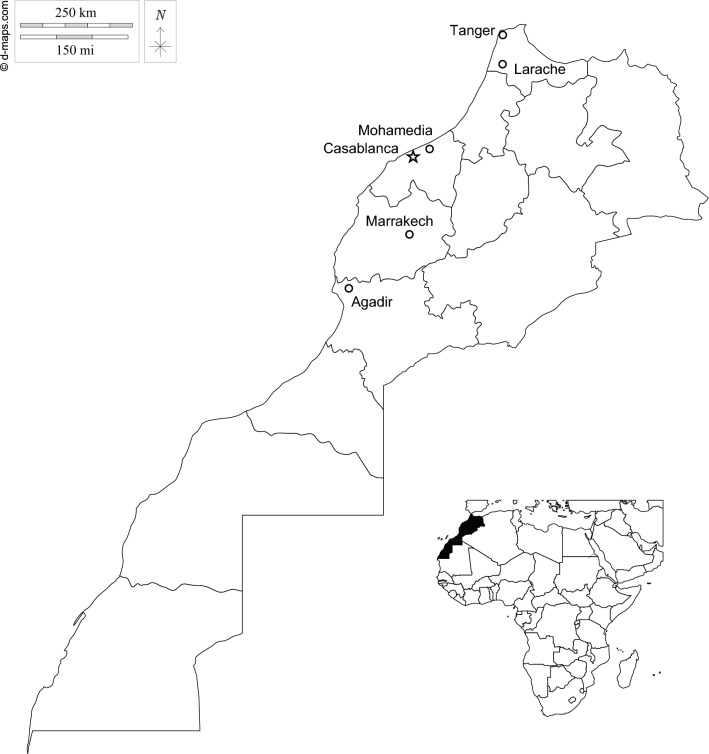
Localities sampled during this study. White dots represented aboveground collection sites; white stars represent underground collection sites

*Culex pipiens* larvae were collected using the dipping sampling method [35] in July 2021 in six regions of Morocco. Various types of breeding sites, either above- or belowground, and different climatic regions representative of the country were sampled (Fig. 1; Table 1). Living larvae were transported to the insectary for identification using the key of Mediterranean Africa mosquitoes [36]: *C. pipiens* larvae were identified based on abdominal characters (a single branch of the caudal seta 1-X, 2–5 branches of the siphon seta 1a-S and no median spine on the segment VIII scales). Only larvae identified as *C. pipiens* were used in subsequent experiments. Samples were collected from densely populated larval sites to minimize the possibility of collecting siblings from the same egg rafts.

**Table 1:** Collection sites of *Culex pipiens* populations sampled in Morocco

Region	Breeding site	Bioclimate	Locality	Latitude	Longitude	Description
Tanger	Aboveground	Humid	Oued Houd	35°46′44.3″N	5°50′50.1″W	Wastewater drain
Larache	Aboveground	Sub-humid	Lokouss	35°10′55.7″N	6°07′56.8″W	Wet meadows
Mohammedia	Aboveground	Sub-arid	Ouled Hmimou	33°40′25.33″N	7°26′42.5″W	Sewer water
Casablanca	Belowground	Sub-arid	Tit mellil	33°34′37.38″N	7°28′23.64″W	Abandoned septic tank
Marrakech	Aboveground	Arid	Souihla	31°38′12.7″N	8°10′07.0″W	Irrigation canals
Agadir	Aboveground	Hyper-arid	Oued Souss	30°21′39.8″N	9°29′22.6″W	Wastewater drain connected to the Souss river

The larvae were provided with a protein-rich diet (1.5 mg protein-rich cat kibble (Friskies® [Nestlé Purina Petcare, St. Louis, MO, USA]) and 0.50 mg of yeast per larva every 2 days) and reared to adults in the laboratory at 28 ± 1 °C, a relative humidity of 80% and a 16:8 h (light:dark) photoperiod [36].

### Autogeny status assessment

**Fig. 2:**
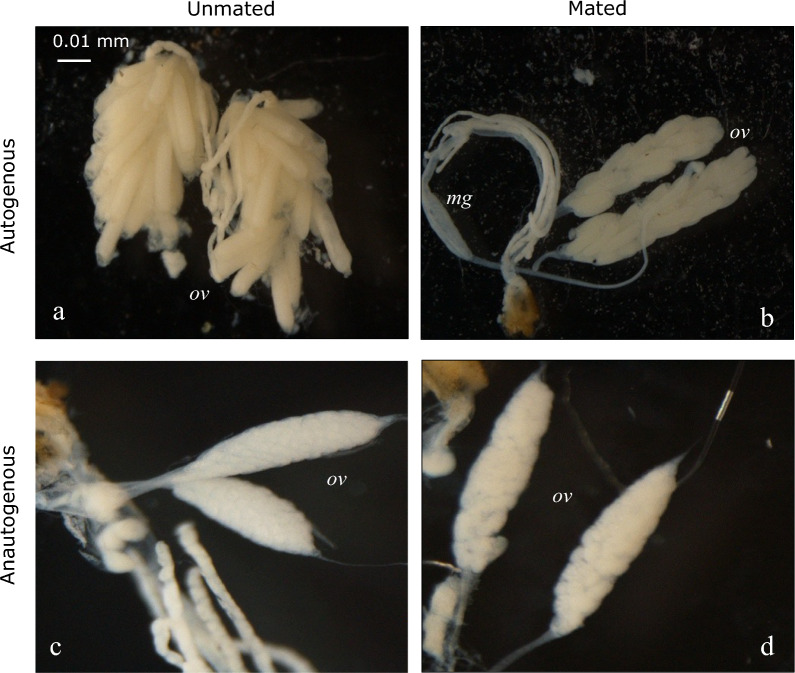
Ovarian development 96 h post-emergence in anautogenous and autogenous *Culex pipiens* females. Photos of the dissected ovaries of different females are presented. Autogenous (**a** unmated, **b** mated) and anautogenous (**c** unmated, **d** mated) females are easily distinguishable when none had access to a blood meal: the follicules in autogenous females are more developed (*i*.*e*. bigger and longer) than those in anautogenous females (*i*.*e*. round and smaller), with mating having only limited visible effects. *mg*, midgut; *ov*, ovary

Females were characterized as autogenous or anautogenous by dissection of their ovaries, following the protocol developed in M.L. Fritz’s laboratory [22]. Briefly, pupae were placed in individual tubes until emergence, then placed in cages containing 10% sucrose, where females were allowed to age for 1 to 4 days. Ovaries were dissected under a stereomicroscope on a Petri dish filled with 90% ethanol. The females were assigned as autogenous or anautogenous directly by visualizing the development of the post-emergence follicles, 96 h after emergence (Fig. 2; note that whether they were able to mate or not before dissection had no effect on the categorization). Classical methods require keeping the females alive for at least 8–10 days, to mate them and let them lay eggs; however, these methods tend to underestimate autogeny frequency (a female laying no eggs can be either anautogenous or simply unwilling to lay eggs in the given laboratory environment). Moreover, females must be isolated when the aim is to associate genotype and autogeny status, which decreases oviposition rate (the presence of specific mosquito oviposition pheromones from other females stimulates oviposition by gravid females; [37]). This dissection-based protocol prevents these biases and constraints, allows the processing of many females, and a rapid and direct assessment of autogeny status and genotype for each individual.

### CQ11 genotyping

DNA was extracted from each genotyped female using the DNAzol method according to the manufacturer's protocol [38]. We determined the CQ11 genotypes of the collected individuals via multiplex PCR tests [27], using the primers pipCQ11R, molCQ11R and CQ11F in a single PCR reaction. The PCR reactions were performed in a 40-μl reaction volume at the following cycling conditions: 30 s at 94 °C; 40 cycles of 30 s at 54 °C and 40 s at 72 °C. Amplified fragments were visualized on a 2% agarose gel: a single DNA fragment of 200 bp corresponds to the *pipiens* form allele, a single DNA fragment of 250 bp corresponds to the *molestus* form allele and individuals displaying both fragments are considered to be heterozygous.

### Data analysis

All computations were performed using the freely available R software (v.4.1.2; http://www.r-project.org; The R Core Team). Departures from Hardy–Weinberg equilibrium were tested for each sample using the *genepop* R package [39]. The correlation between autogeny frequency and CQ11 alleles was analyzed using Fischer exact tests [40].

## Results and discussion

Two forms of *Culex pipiens s*.*s*. are recognized, *pipiens* and *molestus*, described as morphologically identical, but with different behaviors and occupying different habitats [18]. While the distinction between these two forms is clear in northern Europe, it is much less so in North Africa, where the few studies published to date hint at a breakdown of the association between form-specific genetic markers and form-specific behaviors/physiologies, such as autogeny [30–32]. In the present study, this problem was addressed in Morocco, with our question being whether two genetically and behaviorally distinct forms can be found in the southern part of the *C. pipiens s*.*s*. range. More specifically, we collected mosquito larvae from six sites (Fig. 1; Table. 1) that represent Morocco’s five bioclimatic zones and all of the most highly populated geographic areas (large parts of Morocco are deserts), and assessed both the prevalence of autogeny and its local association with the *pipiens/molestus* CQ11 alleles.

### The prevalence of autogeny is variable in Morocco

Larvae identified as *C. pipiens* were reared to adults under laboratory conditions. Females were dissected to analyze their ovaries, as the post-emergence follicle state allows assessment of which females are autogenous (*i*.*e*. able to lay eggs without a blood meal) and which are anautogenous [22]. Figure 2 shows the characteristic aspects of the ovaries of autogenous and anautogenous females (here from the Agadir population). In the absence of a blood meal, the follicules are well developed in autogenous females, whether they are mated of not, but not in anautogenous females; this difference allows an unambiguous characterization. This very efficient autogeny assessment method does not require females to lay eggs and is thus more accurate (*i*.*e*. unbiased and with higher throughput).

Between 100 and 120 females from each population collected were typed. The frequencies of autogeny in the sampled populations are shown in Table 2. Autogeny was found in half of the populations, but at different frequencies, ranging from 1 to 4% in Tangier and Casablanca, to 17.5% in Agadir. Autogeny is thus present across Morocco, in all types of climates (from humid in Tangier to hyper-arid in Agadir).

**Table 2:** Distributions of *Culex pipiens* forms in Morocco

Regions	No. dissected specimens	Autogeny status		CQ11F marker			
		Autogeny status	*N* (%)	No. of genotyped individuals	*pipiens*	*molestus*	heterozygotes
Tanger	100	Autogenous	1 (1%)	1	0 (0.0%)	1 (100.0%)	0 (0.0%)
		Anautogenous	99 (99%)	60	37 (61.6%)	9 (15.0%)	14 (23.3%)
		*Total (%)*		*61*	*60.6%*	*16.3%*	*22.9%*
Larache	100	Autogenous	0 (0%)	–	–	–	–
		Anautogenous	100 (100%)	60	38 (63.3%)	1 (1.6%)	21 (35.0%)
		*Total (%)*		*60*	*63.3%*	*1.6%*	*35.0%*
Mohammedia	100	Autogenous	0 (0%)	–	–	–	–
		Anautogenous	100 (100%)	60	36 (60.0%)	6 (10.0%)	18 (30.0%)
		*Total (%)*		*60*	*60.0%*	*10.0%*	*30.0%*
Casablanca	100	Autogenous	4 (4%)	4	1 (25.0%)	3 (75.0%)	0 (0.0%)
		Anautogenous	96 (96%)	60	34 (56.6%)	6 (10.0%)	20 (33.3%)
		*Total (%)*		*64*	*54.0%*	*14.0%*	*31.2%*
Marrakech	100	Autogenous	0 (0%)	–	–	–	–
		Anautogenous	100 (100%)	60	36 (60%)	8 (13.3%)	16 (26.6%)
		*Total (%)*		*60*	*60.0%*	*13.3%*	*26.6%*
Agadir	120	Autogenous	21 (17.5%)	21	8 (38.0%)	9 (42.8%)	4 (19.0%)
		Anautogenous	99 (82.5%)	99	35 (35.3%)	43 (43.4%)	21 (21.2%)
		*Total (%)*		*120*	*35.8%*	*43.3%*	*20.8%*

The origin of the samples (Regions), the total number of dissected specimens, the autogeny status (autogeneous or anautogeneous) and the percentage of each form are also indicated. The CQ11 microsatellite genotypes were analyzed: the number of genotyped specimens and the number (and percentage) of each genotype are also indicated. Homozygotes for the *pipiens* allele are indicated as *pipiens*, homozygotes for the *molestus* allele are indicated as *molestus* and individuals carrying both alleles are indicated as heterozygote

Autogeny was found in both above- (Tangier and Agadir) and belowground (Casablanca) breeding sites, and the frequency does not appear higher in the belowground versus aboveground populations (although the sampling of belowground populations remains very limited, as they were not found in most sampled areas). These results are similar to those previously obtained in central North Africa: in Tunisia, autogeny was found expressed in aboveground sites with frequencies ranging from 1.43% to 33.3% [30, 32], and autogeny was described in mosquitoes occupying an aboveground habitat in Algeria [31]. However, in these studies, and when quantified, the frequency of autogeny was assessed using crosses in the laboratory, generally in low numbers. The dissection-based method used in the present study allowed processing ≥ 100 females per population, thus providing quantitatively robust and unbiased estimations of autogeny frequency. By contrast, in Russia, northern Europe and northeastern USA, region characterized by a cold climate, autogenous individuals are found exclusively in underground sites [26, 41–43].

### CQ11 allele frequencies are similar across Morocco and across all types of breeding sites

As in previous studies [34, 44, 45], the genotype at the CQ11 locus was used to understand the character of Moroccan populations (e.g.* pipiens*-like, *molestus*-like, intermediate or harboring both discrete types). It should be noted that, as a single locus, CQ11 is not absolutely reliable for individual genotyping; however, it does provide rapid and economical diagnosis of mosquitoes at the population level [27]. Other single-locus methods have been proposed that have proved to be less consistent (see [46]), and while microsatellite markers appear to be more reliable, they are significantly more technical and expensive (e.g. [14]).

As autogeneous females were rare in most populations, we genotyped all autogenous females in all populations, but only 60 anautogenous females out of the 100 characterized for autogeny, except for the Agadir population where autogeny was more prevalent, so that all 120 females were genotyped. The results are presented in Table 2. Both *molestus* and *pipiens* CQ11 alleles were found to be present in all populations, with strikingly similar genotype frequencies: 10–16% *molestus* allele homozygotes (except in the Larache population where it was only approximately 2%), 54–63% *pipiens* allele homozygotes and 23–35% heterozygotes (Table 2). Agadir again appears to be different in terms of genotype frequency, with 43% *molestus* allele homozygotes*,* 36% *pipiens* allele homozygotes and 20% heterozygotes. Overall, this suggests that the climate does not strongly influence CQ11 genotype frequencies, despite the high contrast between the humid north and hyper-arid south environments in Morocco were the different populations were sampled in this study.

The frequencies of the three CQ11 genotypes are similar to what is expected under panmixia in Larache (*F*_is_ = − 0.1213, *P* = 0.67), Mohammedia (*F*_is_ = 0.2081, *P* = 0.16) and Casablanca (*F*_is_ = 0.2092, *P* = 0.12). However, for Agadir (*F*_is_ = 0.6, *p* = 0.000), Marrakech (*F*_is_ = 0.297, *P* = 0.046) and Tanger (*F*_is_ = 0.410, *P* = 0.002), we observed a significant deficit of heterozygotes, which suggests that two populations with more limited gene flow coexist in the same area and tend to lay eggs in the same breeding sites.

Data from previously published studies reporting on the CQ11 genotypes in a natural population of *C. pipiens s*.*s*. in Morocco [34, 45, 47, 48] were re-analyzed to test for departure from panmixia. The results are indicated in Additional file 1: Table S1. They are similar to the results of the present study in that only a few populations showed a significant departure from panmixia. Moreover, it appears that these departures are transient, as for a given locality the results are quite variable, depending on the study and year of sampling (Additional file 1: Table S1).

Finally, the frequency of the *molestus* allele in the belowground site (Casablanca) was found to be similar to that in the aboveground sites (Table. 2). Again, similar results were reported in previously published studies (Additional file 1: Table S1). Although the limited number of populations sampled in the present study does not allow us to make a broad generalization, our results are in accordance with those reported in previous studies from more eastern sites in Algeria and Tunisia, where the *molestus* allele can be found in both above- and belowground habitats [31, 32]. The presence of two discrete forms with genetic and ecological differences is thus less clear in North Africa.

Overall, these results support the notion that *pipiens* and *molestus* may not exist as two discrete forms in Morocco and, more generally, in the North African part of the *C. pipiens *s.s. range, or at least that they cannot be discriminated there using the CQ11 marker [49, 50].

### CQ11 genotype and autogeny appear independent in Morocco

Due to the paucity of autogenous females found in most populations (Table 1), it was only possible to test for an association between CQ11 genotypes and autogeny in the Agadir population. In this aboveground population, > 120 females were analyzed, of which 17% were autogenous, and *pipiens* and *molestus* alleles were found in similar proportions. However, the frequencies of the different CQ11 alleles in autogenous and anautogenous females are strikingly similar (Table 2), and no statistical association between forms and autogeny was found (Fisher’s exact test, *P* = 1), despite evidence for a somewhat structured population at this location (*i*.*e.* a heterozygote deficit; see above). Moreover, the two other populations with a heterozygote deficit (Tanger and Marrakech) did not display high levels of autogeny.

While the results from previous studies from Tunisia [30, 32] suggest this absence of correlation between autogeny and the *molestus* alleles at the aboveground sites (but with a low statistical power, < 20 females per site), the high number of females from the same population analyzed in the present study provides clear support for this lack of correlation. On the southern side of the Mediterranean Sea, there is no association between autogeny and genetic variation at a locus that clearly distinguishes autogenous and anautogenous individuals in regions on the northern side. For example, in Italy, autogeny was found to be totally absent in populations with high *pipiens* allele frequencies [51], and autogeny has similarly been linked to belowground sites and the *molestus* form in the northern part of the *C. pipiens* range [52]. However, other studies found that autogeny is also present in the *pipiens* form in populations from Washington DC [16], and from Portugal (where gene flow between the two forms, although more limited than in Morocco, was evidenced using microsatellites [29]).

Autogeny, usually considered to be a key form-specific capacity, thus appears to be a more labile characteristic than previously thought, and not to be linked to key form-specific genetic variants in North Africa (as well as form-specific ecological preferences for breeding sites). Two alternative hypotheses (not necessarily exclusive) could explain this pattern. First, extensive interbreeding between the two differentiated forms in this region may have homogenized their differences (secondary contact). Second, variable North African populations may represent the ancestral state, with autogeny becoming associated with the *molestus* allele at CQ11 during the differentiation of the two forms elsewhere, through drift and/or selection (differentiation during colonization). Note that this second hypothesis could allow for the undetectable coexistence (at least with currently available diagnostic tools) of the two *molestus* and *pipiens* forms in North African populations. More extensive studies, including a worldwide sampling effort, are required to discriminate the most probable evolutionary scenario.

## Conclusions

In conclusion, we found that there is limited evidence for discrete *pipiens* and *molestus* forms across Morocco: individuals carrying *pipiens* and *molestus* alleles breed in the same sites, with no specific ecology (below- or aboveground sites), and both share the ability to lay eggs without a blood meal (*i.e*. autogeny).

While these observations are fascinating from an evolutionary biology point of view, they are quite worrying in the context of epidemiology. In North Africa, species of the *C. pipiens* complex are considered to be the principal vectors for the transmission of arboviruses, such as West Nile virus (WNV) [53] and Rift Valley Fever virus (RVFV) [54–56]. Both viruses circulate mostly in birds, which are the preferred blood meal source of the *pipiens* form. However, the *molestus* form is usually described as mammophilic. These mixed or intermediate populations could thus act as a bridge vector between mammals and birds [14, 57]. For example, in North America, it has been shown that hybrids between the two forms are actually less discriminating between hosts than pure-form individuals [58]. These mixed or intermediate populations could explain the numerous outbreaks of WNV reported in Morocco: in 1996, 94 equine cases, including 42 deaths, and one human case [59, 60]; in 2003 and 2010, many horses cases reported [61–64]. In addition, the circulation of the WNV was detected in 2018 [65] and confirmed in 2019 by a serological survey in human populations and domestic birds in the northwest of Morocco [66]. It is also possible that host preferences may indeed be shared, potentially increasing pathogen transmission from birds to humans in these regions, and allowing the spread of these characteristics in other parts of the *C. pipiens s*.*s*. complex range. It is therefore urgent that these preferences be investigated in North Africa. Moreover, this is also true for resistance genes: in *C. pipiens s*.*s*. from Morocco, alleles providing resistance to all the classic insecticide families have been found to be associated with all CQ11 allele genotypes [34, 45, 48], which, by easing their spread in the species complex, could complicate the control of the diseases transmitted by this vector.

## Supplementary Information

**Additional file 1: Table S1. **CQ11 genotype data from previously published studies in Morocco. CQ11 genotype data for *C. pipiens s*.s. in Morocco retrieved from the literature are indicated with, for each sample, the locality of sampling, the date (year), the type of breeding site (when reported in the publication, “-” when not), the number of individuals of each CQ11 genotypes (homozygotes for the *pipiens *allele, for the *molestus *allele, and heterozygotes) and the reference. In each case, we tested for departure from panmixia, as for the data of the present study (see Material and Methods): *P*-values < 0.05 are in italics, they are bolded when still significant after sequential Bonferroni correction [67]. As* F*_is_ can be biased for low individual numbers (typically *N *< 30) or low minority allele frequency (typically *f* < 0.05), we only analyzed the samples satisfying these conditions (or at least very close, *N* ≥ 28).

## Data Availability

The datasets supporting the conclusions of this article are included within the article (and its additional file).

## Abbreviations

RVFV: Rift valley fever virus
WNV: West Nile virus

## References

[1] Rivero A, Vezilier J, Weill M, Read AF, Gandon S (2010). Insecticide control of vector-borne diseases: when is insecticide resistance a problem?. PLoS Pathog.

[2] Farajollahi A, Fonseca DM, Kramer LD, Kilpatrick AM (2011). “Bird biting” mosquitoes and human disease: a review of the role of *Culex pipiens* complex mosquitoes in epidemiology. Infect Genet Evol.

[3] Fortuna C, Remoli ME, Di Luca M, Severini F, Toma L, Benedetti E (2015). Experimental studies on comparison of the vector competence of four Italian *Culex pipiens* populations for west nile virus. Parasit Vectors.

[4] Vogels CB, Fros JJ, Göertz GP, Pijlman GP, Koenraadt CJ (2016). Vector competence of northern European *Culex pipiens* biotypes and hybrids for west nile virus is differentially affected by temperature. Parasit Vectors.

[5] Raymond M, Berticat C, Weill M, Pasteur N, Chevillon C. Insecticide resistance in the mosquito *Culex pipiens*: what have we learned about adaptation? Genetica; 2001;112:287–96. 11838771

[6] Hemingway J, Hawkes NJ, McCarroll L, Ranson H (2004). The molecular basis of insecticide resistance in mosquitoes. Insect Biochem Mol Biol.

[7] Hardstone MC, Leichter C, Harrington LC, Kasai S, Tomita T, Scott JG (2007). Cytochrome P450 monooxygenase-mediated permethrin resistance confers limited and larval specific cross-resistance in the southern house mosquito, *Culex pipiens quinquefasciatus*. Pestic Biochem Physiol.

[8] Labbé P, David J-P, Alout H, Milesi P, Djogbenou L, Pasteur N (2017). Evolution of resistance to insecticide in disease vectors evolution of resistance to insecticide in disease vectors.

[9] Milesi P, Weill M, Lenormand T, Labbé P (2017). Heterogeneous gene duplications can be adaptive because they permanently associate overdominant alleles. Evolut lett.

[10] Grigoraki L, Puggioli A, Mavridis K, Douris V, Montanari M, Bellini R (2017). Striking diflubenzuron resistance in *Culex pipiens,* the prime vector of West Nile virus. Sci Rep.

[11] Atyame CM, Labbe P, Dumas E, Milesi P, Charlat S, Fort P (2014). Wolbachia divergence and the evolution of cytoplasmic incompatibility in *Culex pipiens*. PLoS ONE.

[12] Bonneau M, Caputo B, Ligier A, Caparros R, Unal S, Perriat-Sanguinet M (2019). Variation in Wolbachia cidB gene, but not cidA, is associated with cytoplasmic incompatibility mod phenotype diversity in *Culex pipiens*. Mol Ecol.

[13] Vinogradova EB (2000). *Culex pipiens* pipiens mosquitoes: taxonomy, distribution, ecology, physiology, genetics, applied importance and control.

[14] Fonseca DM, Keyghobadi N, Malcolm CA, Mehmet C, Schaffner F, Mogi M (2004). Emerging vectors in the *Culex pipiens* complex. Science.

[15] Harbach RE (2012). *Culex pipiens*: species versus species complex–taxonomic history and perspective. J Am Mosq Control Assoc.

[16] Yurchenko AA, Masri RA, Khrabrova NV, Sibataev AK, Fritz ML, Sharakhova MV (2020). Genomic differentiation and intercontinental population structure of mosquito vectors *Culex pipiens pipien*s and *Culex pipiens molestus*. Sci Rep.

[17] Knight KL (1977). A catalog of the mosquitoes of the world (Diptera: Culicidae). Entomol Soc Am.

[18] Haba Y, McBride L (2022). Origin and status of *Culex pipiens* mosquito ecotypes. Curr Biol.

[19] Harbach RE, Harrison BA, Gad AM (1984). *Culex (Culex) molestus* Forskal (Diptera: Culicidae): neotype designation, description, variation, and taxonomic status. Proc Entomol Soc Wash.

[20] Noriega FG (2004). Nutritional regulation of JH synthesis: a mechanism to control reproductive maturation in mosquitoes?. Insect Biochem Mol Biol.

[21] Dhara A, Eum J-H, Robertson A, Gulia-Nuss M, Vogel KJ, Clark KD (2013). Ovary ecdysteroidogenic hormone functions independently of the insulin receptor in the yellow fever mosquito, *Aedes aegypti*. Insect Biochem Mol Biol.

[22] Jarvela AMC, Bell KL, Noreuil A, Fritz ML (2021). Autogenous and anautogenous *Culex pipiens* bioforms exhibit insulin-like peptide signaling pathway gene expression differences that are not dependent upon larval nutrition. bioRxiv.

[23] Attardo GM, Hansen IA, Raikhel AS (2005). Nutritional regulation of vitellogenesis in mosquitoes: implications for anautogeny. Insect Biochem Mol Biol.

[24] Hansen I, Attardo G, Rodriguez S, Drake L (2014). Four-way regulation of mosquito yolk protein precursor genes by juvenile hormone-, ecdysone-, nutrient-, and insulin-like peptide signaling pathways. Front Physiol.

[25] Gulia-Nuss M, Eum J-H, Strand MR, Brown MR (2012). Ovary ecdysteroidogenic hormone activates egg maturation in the mosquito *Georgecraigius atropalpus* after adult eclosion or a blood meal. J Exp Biol.

[26] Byrne K, Nichols RA (1999). *Culex pipiens* in London Underground tunnels: differentiation between surface and subterranean populations. Heredity.

[27] Bahnck CM, Fonseca DM (2006). Rapid assay to identify the two genetic forms of *Culex* (*Culex*) *pipiens* L. (Diptera: Culicidae) and hybrid populations. Am J Trop Med Hyg.

[28] Chevillon C, Eritja R, Pasteur N, Raymond M (1995). Commensalism, adaptation and gene flow: mosquitoes of the *Culex pipiens* complex in different habitats. Genet Res.

[29] Gomes B, Sousa CA, Novo MT, Freitas FB, Alves R, Côrte-Real AR (2009). Asymmetric introgression between sympatric *molestus* and *pipiens* forms of *Culex pipiens* (Diptera: Culicidae) in the comporta region. Port BMC Evolut Biol.

[30] Krida G, Rhim A, Daaboub J, Failloux A-B, Bouattour A (2015). New evidence for the potential role of *Culex pipiens* mosquitoes in the transmission cycle of west nile virus in Tunisia. Med Vet Entomol.

[31] Korba RA, Alayat MS, Bouiba L, Boudrissa A, Bouslama Z, Boukraa S (2016). Ecological differentiation of members of the *Culex pipiens* complex, potential vectors of West Nile virus and Rift Valley fever virus in Algeria. Parasit Vectors.

[32] Beji M, Rhim A, Roiz D, Bouattour A (2017). Ecophysiological characterization and molecular differentiation of *Culex pipiens* forms (Diptera: Culicidae) in Tunisia. Parasit Vectors.

[33] Knight KL, Abdel Malee AA. A morphological and biological study of *Culex pipiens* in the Cairo area of Egypt (Diptera-Culioidae). Bull Soc Fouad Ent. 1951;35:173–85.

[34] Arich S, Assaid N, Taki H, Weill M, Labbé P, Sarih M (2021). Distribution of insecticide resistance and molecular mechanisms involved in the West Nile vector *Culex pipiens* in Morocco. Pest Manag Sci.

[35] O’Malley C (1995). Seven ways to a successful dipping career. Wing Beats.

[36] Brunhes J, Rhaim A, Geoffroy B, Angel G, Hervy JP. Les culicidae de l’Afrique Méditerranéenne. Un logiciel d’identification et d’enseignement. Montpellier: Institut de Recherche et de Développement; 1999.

[37] Fytrou A, Papachristos DP, Milonas PG, Giatropoulos A, Zographos SE, Michaelakis A (2022). Behavioural response of *Culex pipiens* biotype molestus to oviposition pheromone. J Insect Physiol.

[38] Chomczynski P, Mackey K, Drews R, Wilfinger W (1997). DNAzol^®^: a reagent for the rapid isolation of genomic DNA. Biotech Future Sci.

[39] Rousset F (2008). genepop’007: a complete re-implementation of the genepop software for windows and linux. Mol Ecol Resour.

[40] Presnell B (2000) An introduction to categorical data analysis using r. http://www.stat.ufl.edu/presnell/Courses/sta4504-2000sp/R/R-CDA.pdf. Consulted on February 07, 2022

[41] Vinogradova EB, Shaikevich EV (2007). Morphometric, physiological and molecular characteristics of underground populations of the urban mosquito *Culex pipiens* Linnaeus f. *molestus* Forskål (Diptera: Culicidae) from several areas of Russia. Eur Mosq Bull.

[42] Huang S, Molaei G, Andreadis TG (2008). Genetic insights into the population structure of *Culex pipiens* (Diptera: Culicidae) in the northeastern United States by using microsatellite analysis. Am J Trop Med Hyg.

[43] Becker N, Petric D, Zgomba M, Boase C, Madon M, Dahl C (2010). Mosquitoes and their control.

[44] Amraoui F, Krida G, Bouattour A, Rhim A, Daaboub J, Harrat Z (2012). *Culex pipiens*, an experimental efficient vector of West Nile and rift valley fever viruses in the Maghreb region. PLoS ONE.

[45] Bkhache M, Tmimi F-Z, Charafeddine O, Filali OB, Lemrani M, Labbé P (2019). G119S ace-1 mutation conferring insecticide resistance detected in the *Culex pipiens* complex in Morocco. Pest Manag Sci.

[46] Francuski L, Gojković N, Krtinić B, Milankov V (2019). The diagnostic utility of sequence-based assays for the molecular delimitation of the epidemiologically relevant *Culex pipiens pipiens* taxa (Diptera: Culicidae). Bull Entomol Res.

[47] Amraoui F, Tijane M, Sarih M, Failloux A-B (2012). Molecular evidence of *Culex pipiens* form *molestus* and hybrids *pipiens/molestus* in Morocco, North Africa. Parasit Vectors.

[48] Tmimi F-Z, Faraj C, Bkhache M, Mounaji K, Failloux A-B, Sarih M (2018). Insecticide resistance and target site mutations (G119S ace-1 and L1014F kdr) of *Culex pipiens* in Morocco. Parasit Vectors.

[49] Roubaud É. Le Pouvoir autogène chez le biotype nord-africain du moustique commun, "*Culex pipiens*" L. Paris: Masson; 1939.

[50] Rioux JA, Juminer B, Kchouk M, Croset H (1965). Présence du caractère autogène chez *Culex pipiens*
*pipiens* L dans un biotope épigé de l’Ile de Djerba. Arch Inst Pasteur Tunis.

[51] Di Luca M, Toma L, Boccolini D, Severini F, La Rosa G, Minelli G (2016). Ecological distribution and CQ11 genetic structure of *Culex pipiens* complex (Diptera: Culicidae) in Italy. PLoS ONE.

[52] Roubaud E. Autogenous cycle of winter generations of *Culex pipiens* L. C R Acad Sci. 1929;188:735–38.

[53] Krida G, Diancourt L, Bouattour A, Rhim A, Chermiti B, Failloux AB (1990). Assessment of the risk of introduction to Tunisia of the rift valley fever virus by the mosquito *Culex Pipiens*. Bull Soc Pathol Exot.

[54] Meegan JM, Khalil GM, Hoogstraal H, Adham FK (1980). Experimental transmission and field isolation studies implicating *Culex pipiens* as a vector of rift valley fever virus in Egypt. Am J Trop Med Hyg.

[55] Hoogstraal H, Meegan JM, Khalil GM, Adham FK (1979). The rift valley fever epizootic in Egypt 1977–78 2 ecological and entomological studies. Trans R. Soc Trop Med Hyg.

[56] Moutailler S, Krida G, Schaffner F, Vazeille M, Failloux A-B (2008). Potential vectors of Rift Valley fever virus in the Mediterranean region. Vector-borne Zoonotic Dis.

[57] Hamer GL, Kitron UD, Brawn JD, Loss SR, Ruiz MO, Goldberg TL (2008). *Culex pipiens* (Diptera: Culicidae): a bridge vector of West Nile virus to humans. J Med Entomol.

[58] Fritz ML, Walker ED, Miller JR, Severson DW, Dworkin I (2015). Divergent host preferences of above-and below-ground *Culex pipiens* mosquitoes and their hybrid offspring. Med Vet Entomol.

[59] Tber AA. West nile fever in horses in Morocco. Bull Off Intern Epizoot. 1996;108:867–9.

[60] El Harrack M, Le Guenno B, Gounon P (1997). Isolement du virus West Nile au Maroc. Virologie.

[61] Schuffenecker I, Peyrefitte CN, El Harrak M, Murri S, Leblond A, Zeller HG (2005). West Nile virus in Morocco 2003. Emerg Infect Dis.

[62] El Rhaffouli H, El Harrak M, Lotfi C, El Boukhrissi F, Bajjou T, Laraqui A (2012). Serologic evidence of West Nile virus infection among humans Morocco. Emerg Infect Dis.

[63] Durand B, Haskouri H, Lowenski S, Vachiéry N, Beck C, Lecollinet S (2016). Seroprevalence of West Nile and Usutu viruses in military working horses and dogs, Morocco, 2012: dog as an alternative WNV sentinel species?. Epidemiol Infect.

[64] Benjelloun A, El Harrak M, Calistri P, Loutfi C, Kabbaj H, Conte A (2017). Seroprevalence of West Nile virus in horses in different Moroccan regions. Vet Med Sci.

[65] Assaid N, Mousson L, Moutailler S, Arich S, Akarid K, Monier M (2020). Evidence of circulation of West Nile virus in *Culex pipiens* mosquitoes and horses in Morocco. Acta Trop.

[66] Assaid N, Arich S, Ezzikouri S, Benjelloun S, Dia M, Faye O (2021). Serological evidence of West Nile virus infection in human populations and domestic birds in the northwest of Morocco. Comp Immunol Microbiol Infect Dis.

[67] Hochberg Y (1988). A sharper bonferroni procedure for multiple tests of significance. Biometrika.

