# Supplementary material for: No association between habitat, autogeny and genetics in Moroccan Culex pipiens populations
Source: Parasit Vectors. 2022 Nov 3;15:405. doi: 10.1186/s13071-022-05469-3 (PMC9635193; doi:10.1186/s13071-022-05469-3)
Supplement: Supplementary file 1 — Additional file 1: Table S1. CQ11 genotype data from previously published studies in Morocco. CQ11 genotype data for C. pipiens s.s. in Morocco retrieved from the literature are indicated with, for each sample, the locality of sampling, the date (year), the type of breeding site (when reported in the publication, “-” when not), the number of individuals of each CQ11 genotypes (homozygotes for the pipiens allele, for the molestus allele, and heterozygotes) and the reference. In each case, we tested for departure from panmixia, as for the data of the present study (see Material and Methods): P-values < 0.05 are in italics, they are bolded when still significant after sequential Bonferroni correction [67]. As Fis can be biased for low individual numbers (typically N < 30) or low minority allele frequency (typically f < 0.05), we only analyzed the samples satisfying these conditions (or at least very close, N ≥ 28). [file 13071_2022_5469_MOESM1_ESM.pdf]

Additional file 1

Table S1: CQ11 genotype data from previously published studies in Morocco. CQ11 genotype data for *C. pipiens s.s.* in Morocco retrieved from the literature are indicated with, for each sample, the locality of sampling, the date (year), the type of breeding site (when reported in the publication, "-" when not), the number of individuals of each CQ11 genotypes (homozygotes for the *pipiens* allele, for the *molestus* allele and heterozygotes), and the reference. In each case, we tested for departure from panmixia, as for the data of the present study (see Material and Methods): *p*-values < 0.05 are in italics, they are bolded when still significant after sequential Bonferroni correction (Hochberg, 1988). As F_{is} can be biased for low individuals numbers (typically N <30) or low minority allele frequency (typically f <0.05) [ref], we only analyzed the samples satisfying these conditions (or at least very close, N ≥28).

[1] In this publication, a single population was sampled, sub-samples being tested for different insecticides. The numbers indicated here are the sums of all sub-samples.

Bibliography:

Amraoui, F., Tijane, M., Sarih, M., Failloux, A.-B. Molecular evidence of *Culex pipiens* form *molestus* and hybrids *pipiens*/*molestus* in Morocco, North Africa. Parasites & Vectors 5, 83.

Arich, S., Assaid, N., Taki, H., Weill, M., Labbé, P., Sarih, M., 2021. Distribution of insecticide resistance and molecular mechanisms involved in the West Nile vector *Culex pipiens* in Morocco. Pest Management Science. 2012;77, 1178–1186.

Bkhache, M., Tmimi, F.-Z., Charafeddine, O., Faraj, C., Failloux, A.-B., Sarih, M.. First report of L1014F-*kdr* mutation in *Culex pipiens* complex from Morocco. Parasites & vectors. 2016;9, 644.

Hochberg, Y. A sharper Bonferroni procedure for multiple tests of significance. Biometrika. 1988;75, 800–802.

Tmimi, F.-Z., Faraj, C., Bkhache, M., Mounaji, K., Failloux, A.-B., Sarih, M. Insecticide resistance and target site mutations (G119S *ace-1* and L1014F *kdr*) of *Culex pipiens* in Morocco. Parasites & vectors. 2018;11, 51.

Locality	Year	Breeding site	*pipiens*	*molestus*	heterozygotes	*Fis*	*P-value*	Reference
**Tanger**	2015	-	48	2	37	-0.179	0.13	Bkhache et al., 2016
	2015	-	45	1	22	-0.106	0.67	Bkhache et al., 2016
	2018	Aboveground	38	1	19	-0.096	0.66	Arich et al., 2021
**Larache**	2018	Aboveground	25	4	31	-0.196	0.24	Arich et al., 2021
**Mohammedia**	***2010***	***Belowground***	***8***	***19***	***5***	***0.655***	***0.0004***	Amraoui et al., 2012
	2010	Aboveground	15	5	8	0.361	0.08	Amraoui et al., 2012
	2016	Aboveground	299	48	186	0.066	0.16	Tmimi et al., 20181
	2018	Aboveground	27	7	24	0.069	0.76	Arich et al., 2021
**Casablanca**	2010	Aboveground	9	5	15	-0.037	1	Amraoui et al., 2012
	2015	***-***	***38***	***80***	***36***	***0.546***	***0***	Bkhache et al., 2016
	2015	-	35	2	5	0.388	0.05	Bkhache et al., 2016
**Marrakech**	2015	-	26	0	18	-0.246	0.16	Bkhache et al., 2016
	2015	-	20	9	20	-0.012	1	Bkhache et al., 2016
	2018	Aboveground	18	10	32	-0.078	0.6	Arich et al., 2021
**Agadir**	2018	*Aboveground*	*11*	*9*	*37*	*-0.292*	*0.03*	Arich et al., 2021
